# RPS24 Is Associated with a Poor Prognosis and Immune Infiltration in Hepatocellular Carcinoma

**DOI:** 10.3390/ijms24010806

**Published:** 2023-01-02

**Authors:** Haiyuan Li, Lei Gao, Xiaojuan Kang, Xueyan Wang, Yang Yu, Yaqing Zhang, Hao Chen

**Affiliations:** 1 Second Clinical Medical College, Lanzhou University, Lanzhou 730000, China; 2 Department of Tumor Surgery, Lanzhou University Second Hospital, Lanzhou 730030, China

**Keywords:** RPS24, hepatocellular carcinoma, prognosis, immune infiltration, cell proliferation

## Abstract

Hepatocellular carcinoma (HCC) is the most common type of primary liver malignancy, with increased mortality and morbidity. Accumulating evidence suggested that 40S ribosomal protein S24 (RPS24) is related to malignant outcomes and progression. However, the role of RPS24 remains unclear in HCC. The mRNA and protein expression pattern of RPS24 in HCC was explored and confirmed based on the bioinformatics analysis and histological examination. The correlation between RPS24 expression and clinicopathological features, diagnostic value, prognosis, methylation status, and survival were evaluated. Then, we divided the HCC cohort into two groups based on the expression of RPS24, and performed the functional enrichment and immune cells infiltration analysis of RPS24. Furthermore, in vivo and in vitro experiments were performed to investigate the effect of RPS24 on HCC cells. RPS24 was observed to be elevated in HCC samples. RPS24 overexpression or RPS24 promoter methylation contributed to an unfavorable prognosis for HCC patients. The genes in the high RPS24 expression group were mainly enriched in DNA replication, cell cycle E2F targets, and the G2M checkpoint pathway. Moreover, the expression level of RPS24 was significantly related to immune infiltration and immunotherapy response. Our experiments also demonstrated that RPS24 knockdown suppressed the growth of HCC cells and tumor proliferation of the xenograft model. Therefore, RPS24 can be a potential adverse biomarker of HCC prognosis acting through facilitating cell proliferation and the formation of an immunosuppressive microenvironment in HCC. Targeting RPS24 may offer a promising therapeutic option for HCC management.

## 1. Introduction

Hepatocellular carcinoma (HCC), with its increasing incidence rate, remains a severe threat to public health [1,2,3]. Although the clinical treatment of HCC and development of drugs for the disease have contributed to dramatic progress, many patients experience tumor recurrence or progression following radical HCC surgery, drug therapy, or immunotherapy [4]. In addition, patients are often in the middle and late stages when diagnosed, which results in a poor prognosis [5,6]. Although many studies were conducted on the mechanisms of HCC, the genes with cancer-promoting signatures remain unclear. Therefore, the identification of new targets or biomarkers and elucidation of the underlying tumor-driving mechanisms are urgently required.

The eukaryotic ribosome is a complex enzyme system composed of four ribosomal RNAs (rRNAs) and 80 ribosomal proteins (RPs) [7,8]. Studies confirmed that RP is involved in protein synthesis and crucial for the hallmarks of tumors, such as DNA damage repair, the tumor cell cycle, cell division, apoptosis, tumor metastasis, and tumor drug resistance [8,9,10,11]. The 40S ribosomal protein S24 (RPS24), one of the ribosomal proteins, significantly drives tumor occurrence and development. Arthurs et al. found that RPS24 was highly expressed in 82 prostate cancer tissues, suggesting that RPS24 could act as a diagnostic biomarker [12]. Kazerounian et al. reported that RPS24 is an oncoprotein of solid cancers [13]. Moreover, a recent study reported that the inhibition of RPS24 expression with small hairpin shRNA can suppress cell proliferation and migration in colon cancer [14]. However, the question of whether or not RPS24 regulates HCC development remains obscure.

This study first examined RPS24 expression in HCC and its correlation with the clinicopathological features of the disease based on the Cancer Genome Atlas (TCGA) database and clinical samples. The enriched signaling pathways and methylation status of RPS24 in HCC were investigated. Since immune status is critical for the survival of HCC patients [15,16], the relationship between RPS24 expression and immune cell infiltration, immune checkpoints, and the immunotherapy response was examined. Moreover, the biological functions of RPS24 were explored in HCC cells in vivo and in vitro.

## 2. Results

### 2.1. RPS24 Was Predominantly Up-Regulated in HCC

**Figure 1:**
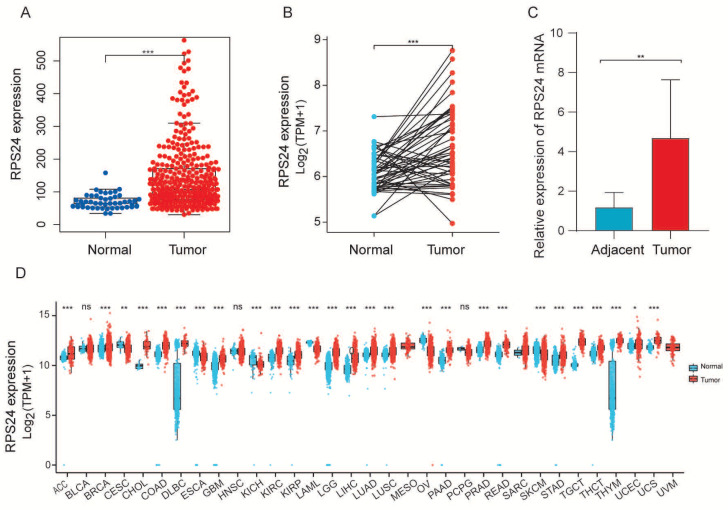
RPS24 is highly expressed in HCC. (**A**,**B**) Detection of RPS24 mRNA in cancerous samples with unpaired (**A**) and paired normal tissues using the TCGA-LIHC dataset (**B**). (**C**) In 20 paired samples of HCC patients, the RPS24 mRNA levels were tested using qRT-PCR (cohort 1). (**D**) The expression of RPS24 mRNA in pan-cancers using the TCGA tumor database. ns *p* ≥ 0.05; * *p* < 0.05;** *p* < 0.01; *** *p* < 0.001.

To evaluate the role of RPS24 in HCC development, we first estimated the mRNA level of RPS24 in the TCGA database and found that RPS24 was observably up-regulated in HCC compared with normal liver tissue (Figure 1A,B). qRT-PCR confirmed that RPS24 expression in HCC tissues was observably higher than that in the adjacent non-tumor tissues (cohort 1) (Figure 1C). Furthermore, 28 types of tumors were examined for the mRNA level of RPS24, showing that RPS24 was elevated in most of the tumors, such as cholangiocarcinoma (CHOL), thyroid carcinoma (THCA), rectum adenocarcinoma (READ), and thymoma (THYM) (Figure 1D).

### 2.2. RPS24 Expression Was Associated with the Clinicopathologic Parameters

**Figure 2:**
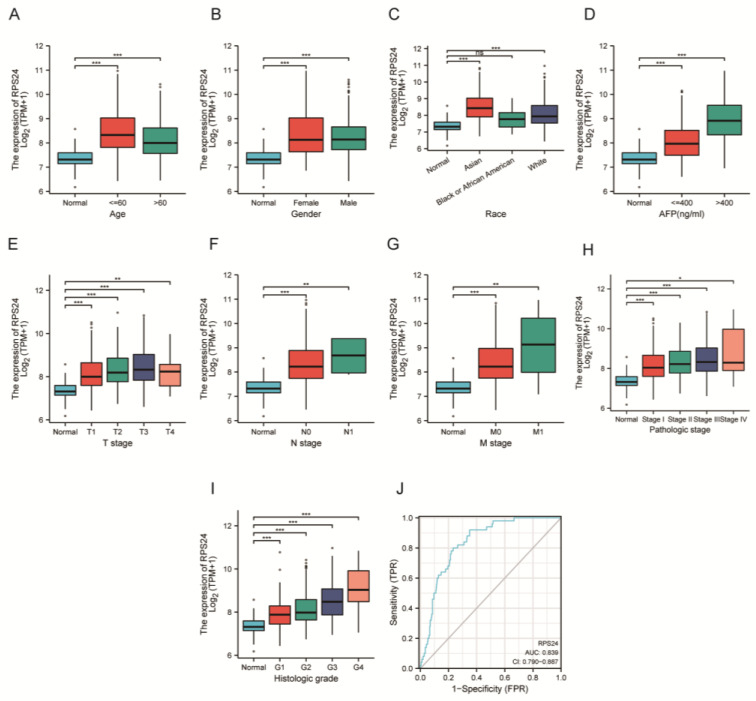
RPS24 mRNA expression in patients with different clinicopathological features. (**A**–**I**) Comparison of RPS24 expression in normal liver tissue and cancer tissue with various clinicopathological features, including age, sex, pathological stage, lymph node metastasis, distant metastasis, histological grade, race, and AFP was performed using the TCGA-LIHC dataset. (**J**) The diagnostic value of RPS24 expression in normal individuals and cancer patients was performed using the TCGA-LIHC dataset. ns *p*≥0.05; * *p* < 0.05; ** *p* < 0.01; *** *p* < 0.001.

Cancer progression and treatment efficacy are known to be impacted by different clinicopathologic parameters [1,17]. Next, we analyzed the correlation between the clinicopathological parameters of HCC and RPS24 expression in the TCGA database, showing that the RPS24 mRNA levels were significantly higher in HCC patients than in the controls in regard to age, sex, the pathological stage, lymph node metastasis, distant metastasis, histological grade, race, and alpha fetoprotein (AFP) (Figure 2A–I). A logistic regression analysis showed that the RPS24 mRNA levels were higher in patients with grades G3 and G4 than in patients with grades G1 and G2 (*p* < 0.001), in patients with AFP > 400 ng/mL than in patients with AFP ≤ 400 ng/mL (*p* < 0.001), and in patients aged > 60 years than in patients aged ≤ 60 years (*p* = 0.026) (Table 1). In addition, we evaluated the diagnostic value of RPS24 expression for HCC patients using ROC curves. The results showed an area under the curve (AUC) of 0.839 for RPS24 (Figure 2J). Subsequently, we assessed the AUC values of RPS24 expression in stages I and II, III and IV, G1 and G2, G3 and G4, R0, and R1 and R2, which were 0.827, 0.889, 0.805, 0.894, 0.837, and 0.919, respectively (Figure S1), indicating that the more severe the disease is, the higher the diagnostic value will be.

### 2.3. The Overexpression of RPS24 Led to a Poor Prognosis for HCC Patients

**Figure 3:**
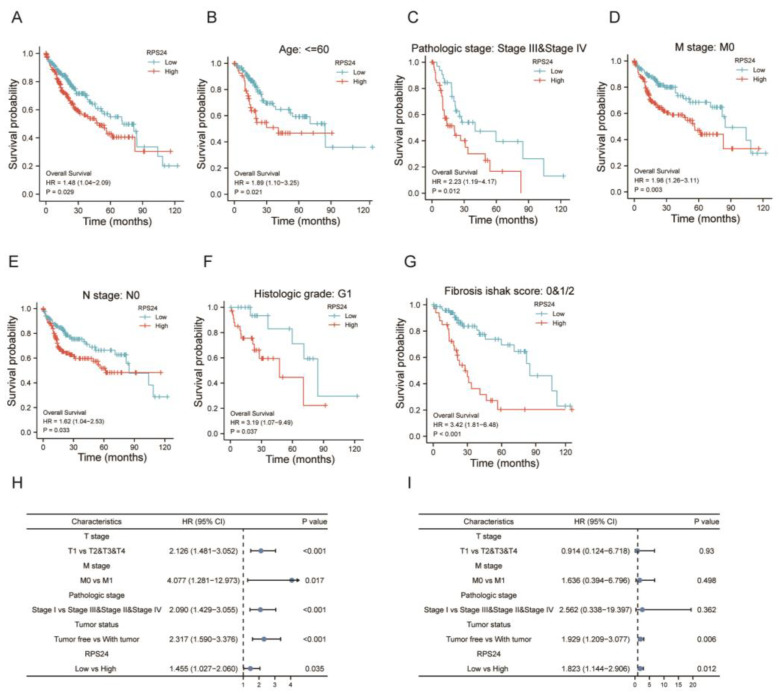
High expression of RPS24 and poor prognosis in HCC. (**A**) The OS survival curve between high and low expressions of RPS24 was drawn using the TCGA-LIHC dataset. (**B**–**G**) The OS survival curves of HCC between high and low expressions of RPS24 in subgroups according to age of ≤ 60 years (**B**), pathological stages III and IV (**C**), M0 (**D**), N0 (**E**), G1 grade (**F**), and Fibrosis ishak score: 0&1/2 (**G**). (**H**,**I**) Forest plot showing univariate (**H**) and multivariate analyses (**I**) of RPS24 in the TCGA-LIHC data.

As shown in Figure 3A, the overall survival (OS) of patients with high RPS24 expression was remarkably shorter than that of patients with low RPS24 expression (*p =* 0.029). A subgroup analysis showed that the overexpression of RPS24 significantly affected the OS of HCC patients aged ≤ 60 years (*p* = 0.021) with pathological stages III and IV (*p* = 0.012), M0 (*p* = 0.003), N0 (*p* = 0.033), G1 grade (*p* = 0.037), and a fibrosis ishak score of 0&1/2 (*p* < 0.001) (Figure 3B–G). A univariate analysis showed that RPS24, the T stage, M stage, pathological stage, and tumor status were associated with OS (Figure 3H). Based on our multivariate analysis, we observed that RPS24 was an independent risk predictor for HCC patients (Figure 3).

### 2.4. Methylation Analyses of RPS24 Expression in HCC

**Figure 4:**
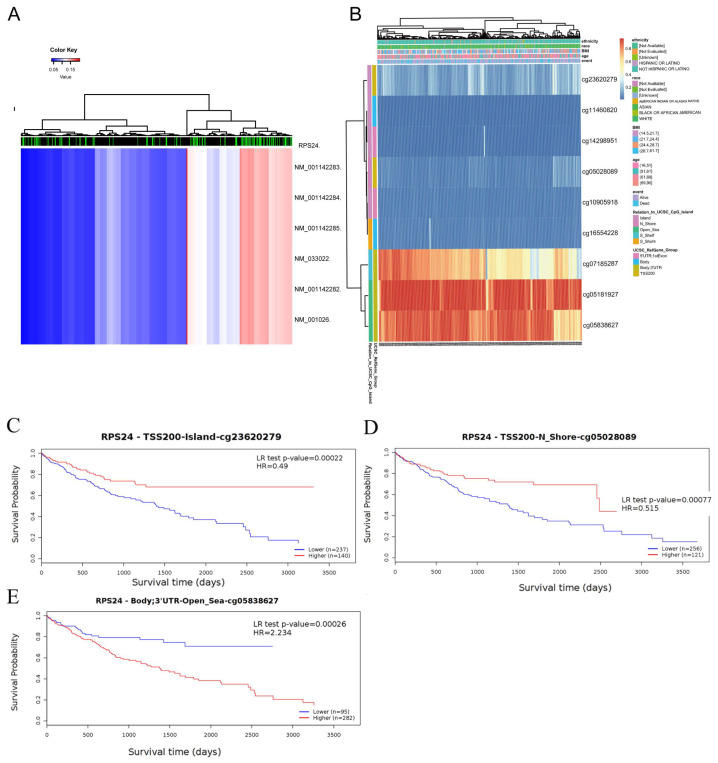
Methylation analyses of RPS24 expression in HCC. (**A**) We used the online DiseaseMeth database to compare the methylation levels of the RPS24 promoter in HCC cancer tissues and normal liver tissues. Each column represents a sample, with green representing normal samples and black representing tumor samples. The redder the heat map color is, the higher the methylation level is (**B**). Heatmap of methylation changes at multiple sites in the RPS24 DNA sequence, analyzed using the Methsurv database. (**C**–**E**) The relationship between methylation and prognosis at different loci: cg05028089 (**C**), cg05838627 (**D**), and cg23620279 (**E**).

In addition, we analyzed the RPS24 methylation status using the online DiseaseMeth database. We found that the level of DNA methylation on the promoters of different transcripts of RPS24 in HCC tissues was remarkably lower than that in normal tissues (*p* < 0.01) (Figure 4A, Table S1). Meanwhile, using the Methsurv database, we identified methylation changes at multiple sites in the RPS24 DNA sequence (Figure 4B) which were associated with a poor prognosis, including cg05028089 (Figure 4C), cg05838627 (Figure 4D), and cg23620279 (Figure 4E).

### 2.5. Functional Analysis and Micro-(mi)RNA-Regulated Network Analysis of RPS24 in HCC

**Figure 5:**
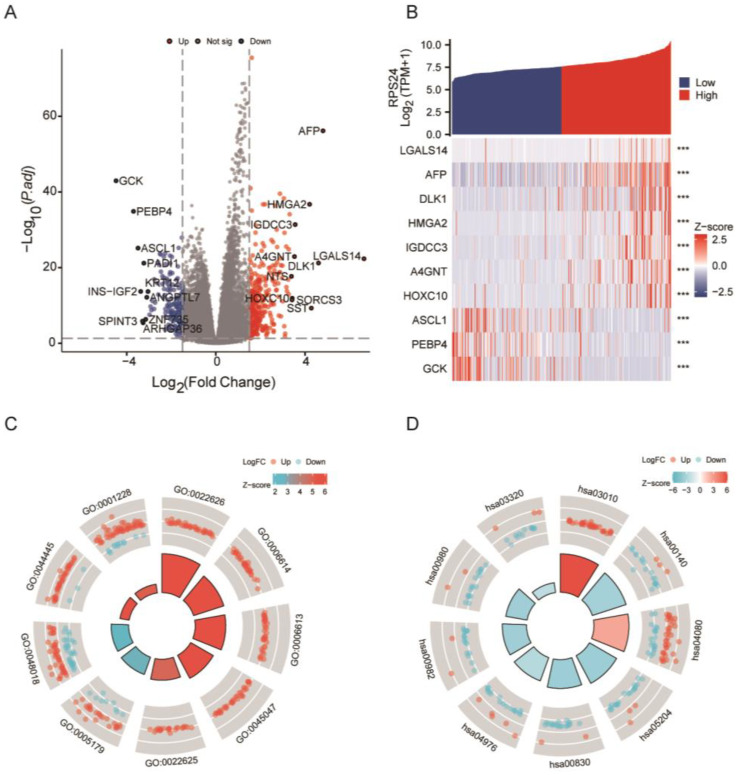
Identification of DEGs and PPI network Analysis. (**A**)Volcano plot of the DEGs of RPS24, depicting the top 10 up- and down-regulated DEGs. (**B**) The relationship between the top 10 DEGs (including LGALS14, DLK1, AFP, HMGA2, IGDCC3, A4GNT, HOXC10, ASCL1, PEBP4, and GCK) and RPS24 was analyzed. (**C**,**D**) GO and KEGG pathway enrichment analyses of the DEGs of RPS24 were performed. *** *p* < 0.001.

**Figure 6:**
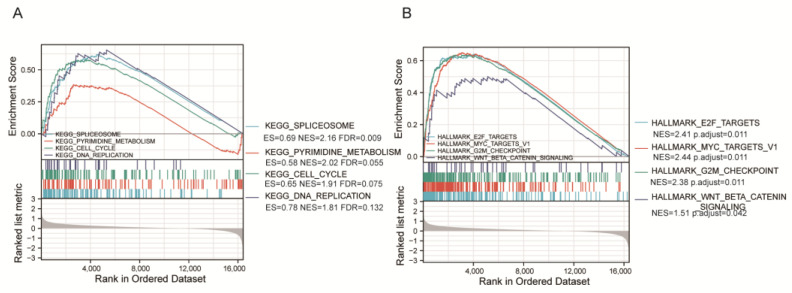
GSEA analyses of RPS24 expression in LIHC. (**A**)The four KEGG signaling pathways were markedly enriched with the high expression of RPS24, including the spliceosome, pyrimidine metabolism, cell cycle, and DNA replication. (**B**)The four HALLMARK pathways were significantly enriched with the high expression of RPS24, including the E2F targets, MYC targets, G2M-checkpoint-related pathways, and Wnt/β-catenin signaling.2.6. Immune infiltration analysis of RPS24 in HCC.

To further elucidate the role of RPS24 in HCC, we identified DEGs based on the expression of RPS24. The results indicated that 371 were up-regulated DEGs (67.09%) and 182 were down-regulated DEGs (32.91%). (Figure 5A). Subsequently, we examined the relationship between the top 10 DEGs and RPS24 (Figure 5B) and found that seven key genes were positively correlated with RPS24. As presented in Figure 5C, the DEGs were mainly enriched in hormone activity, receptor ligand activity, DNA-binding transcription activator activity, and RNA polymerase II-specific activity in the RPS24-high expression group. The results of the KEGG analysis demonstrated that the DEGs were mainly enriched in ribosome, steroid hormone biosynthesis, neuroactive ligand–receptor interaction, and chemical carcinogenesis (Figure 5D, Table S2). We further conducted a GSEA analysis using the KEGG pathway gene sets and HALLMARK gene sets. In the case of the KEGG pathways, the spliceosome, pyrimidine metabolism, cell cycle, and DNA replication were markedly enriched with the high expression of RPS24 (Figure 6A,B). In the case of the HALLMARK pathways, a high expression of RPS24 was associated with the activation of cancer-promoting pathways, especially in regard to cell proliferation and immune-related signaling pathways (Figure S2). In addition, an investigative analysis of the network interaction between RPS24 and miRNA revealed that hsa-miR-4267, hsa-miR-4435, hsa-miR-4435, hsa-miR-4435, hsa-miR-93-3p, hsa-miR-204-3p, hsa-miR-373-5p, hsa-miR-3192-5p, hsa-miR-3192-5p, hsa-miR-4267, hsa-miR-4447, hsa-miR-4516, hsa-miR-4701-3p, hsa-miR-3064-5p, hsa-miR-3064-5p, hsa-miR-4750-3p, hsa-miR-6504-5p, hsa-miR-6763-5p, hsa-miR-6880-5p, hsa-miR-8077, and hsa-miR-9899 are co-expressed in HCC development (Figure S3).

**Figure 7:**
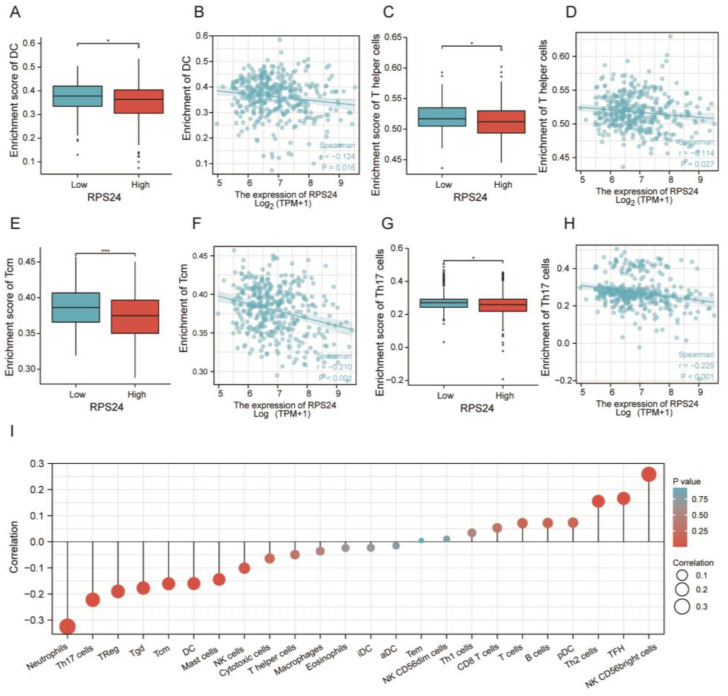
Correlation of RPS24 with immune cell infiltration. (**A**–**H**) RPS24 expression was negatively correlated with DC, T helper cells, Tcm, and Th17 cells based on the TCGA-LIHC data. (**I**) The lollipop showed that RPS24 was correlated with several infiltrated immune cells. Abbreviations: DC, dendritic cell; Tem, effective memory T-cell. * *p* < 0.05; *** *p* < 0.001.

**Figure 8:**
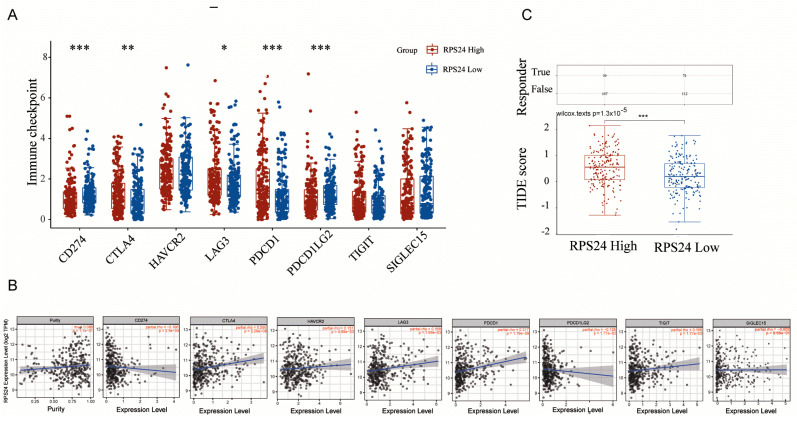
Correlation of RPS24 with immune checkpoint molecules and immunotherapy response. (**A**) The immune checkpoint molecules were measured in HCC patients in the low- and high-RPS24 groups using the TCGA-LIHC data. (**B**) The relationship between RPS24 expression and immune checkpoint molecules was measured using the TIMER database. (**C**) TIDE scores were measured in HCC patients in the low- and high-RPS24 groups using the TCGA-LIHC data. * *p* < 0.05; ** *p* < 0.01; *** *p* < 0.001.

To assess whether RPS24 affected the infiltration of immune cells, we performed a correlation analysis, showing that RPS24 expression was negatively correlated with the infiltration level of some immune cells, such as DC, T helper cells, Tcm, and Th17 cells (Figure 7A–H), which were consistent with the lollipop (Figure 7I). Moreover, the correlation between RPS24 and immune checkpoint molecules was investigated, showing that CTLA4, HAVCR2, LAG3, and PDCD1 were significantly increased in the high-RPS24-expressed group, whereas CD274 and PDCD1LG2 were considerably decreased (Figure 8A), findings which were consistent with the analysis of the TIMER database (Figure 8B). Furthermore, the immunotherapy response was assessed using the TIDE scores and compared between the high- and low-RPS24-expression groups, showing that patients with high RPS24 expression exhibited higher TIDE scores (a low response probability) (Figure 8C). The abovementioned findings suggest that the increased expression of RPS24 might enhance the immune evasion of HCC and have high accuracy in identifying highly immune-responsive patients.

### 2.6. Validation of RPS24 Expression in HCC Tissues

**Figure 9:**
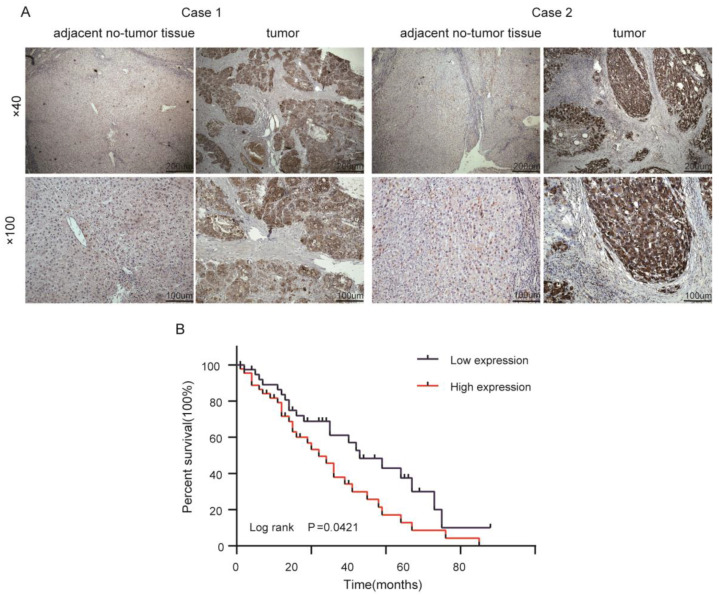
High expression of RPS24 and poor prognosis in HCC. (**A**) Immunohistochemical staining of RPS24 levels was performed using 86 pairs of cancer tissue and adjacent non-tumor tissue, and representative images are shown. (**B**) The survival analysis was performed on different RPS24 groups using cohort 2.

Next, we tested the expression of the RPS24 protein in 86 pairs of HCC and adjacent non-tumor tissues (cohort 2) using IHC and showed that RPS24 proteins have different degrees of staining in the cytoplasm, and the protein expression of RPS24 in HCC was significantly higher than that in the non-tumor tissues (Figure 9A, Table 2), suggesting that RPS24 has a cancer-promoting effect on the cytoplasm of HCC cells. This finding was consistent with the results of cohort 1 and the TCGA database.

To investigate the clinical effects of RPS24 on patients with hepatocellular carcinoma, we classified the HCC patients into a “high group” (*n* = 45) and “low group” (*n* = 41) based on the IHC staining indicators of RPS24 expression. It was found that high TF was closely connected to AFP (*p* = 0.033) and the tumor size (*p* = 0.034) and number (*p* = 0.020) (Table 3). Subsequently, Kaplan–Meier survival analysis was carried out to elucidate the relationship between RPS24 expression and OS in the 86 HCC patients. Our results revealed that the high group had a shorter OS than the low group (*p* = 0.0421) (Figure 9B).

### 2.7. Knockdown of RPS24 Inhibited the Proliferation of HCC Cells

**Figure 10:**
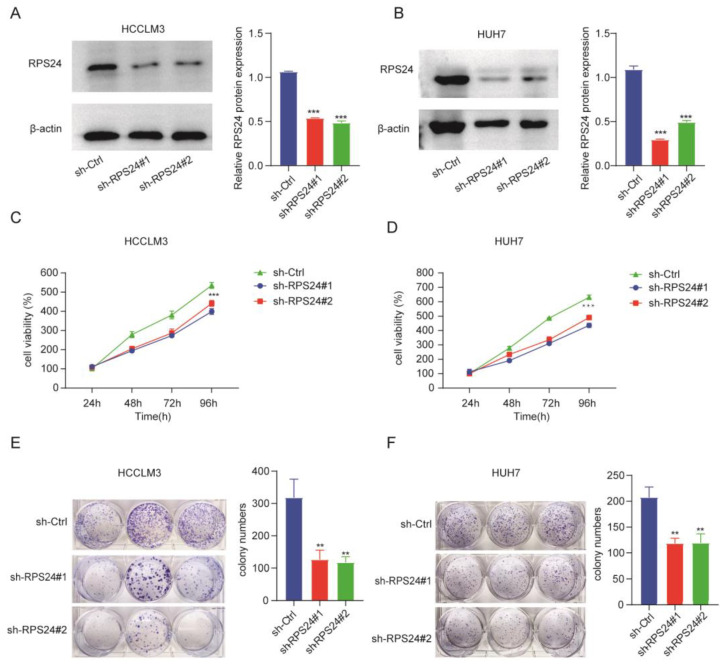
Knockdown of RPS24 impeded the cell proliferation of HCC. (**A**,**B**) The protein levels of RPS24 in the sh-RPS24 group and the sh-Ctrl group were tested using Western blot analysis. The CC-K8 assay displayed the effect of RPS24 knockdown on cell proliferation in the HCCLM3 (**C**) and HuH-7 (**D**) cell lines. (**E**,**F**) Colony formation capabilities of HCCLM3 (**E**) and HuH-7 (**F**) after RPS24 knockdown cells are shown. ** *p* < 0.01; *** *p* < 0.001.

To further verify the effect of RPS24 on cell proliferation, we established stable cell lines with lentivirus, targeting RPS24 in the HCCLM3 and HUH-7 cell lines (Figure 10A,B). Next, the cell proliferation in the sh-RPS24 group and the control group was evaluated through the CCK-8 experiment, and the results indicated that the ability of the HCCLM3 and HUH-7 cells to proliferate was dramatically inhibited following transfection with RPS24-shRNA (Figure 10C,D). In addition, the colony formation assay confirmed the negative regulation of the cells’ proliferation ability after their transfection with RPS24-shRNA (Figure 10E,F). These data suggest that RPS24 knockdown impedes the proliferation of HCC cell lines.

### 2.8. Knockdown of RPS24 Reduced the Growth of Tumor Xenografts in Mice

**Figure 11:**
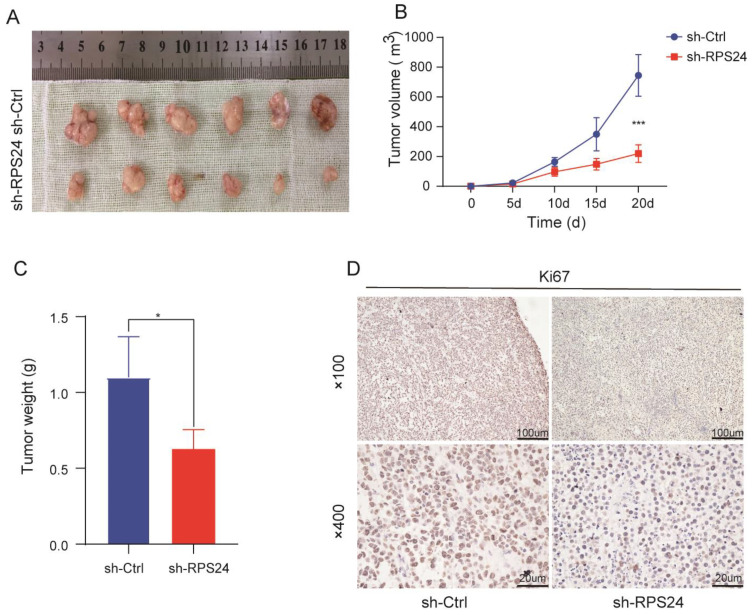
Knockdown of RPS24 reduced tumor growth in mice. Subcutaneous tumor samples (**A**), tumor growth curves (**B**), and tumor weight (**C**) of the sh-RPS24 group and the sh-Ctrl group after the transfected HCCLM3 cells were inoculated in SCID mice for 20 days are presented. (**D**) Immunohistochemical staining of the Ki67 levels in the tumor tissues was performed on both groups. * *p* < 0.05; *** *p* < 0.001.

To evaluate the effect of RPS24 on tumor growth in vivo, HCCLM3 cells lacking RPS24 were used to establish a subcutaneous xenograft model of mice. We found that the tumor weight and volume in the sh-RPS24 group were substantially lower than those in the sh-Ctrl group (Figure 11A–C). In addition, the expression level of Ki67 was detected by immunohistochemistry. Decreased Ki67 was detected in tumors of the RPS24 knockdown group (Figure 11D). These results suggest that RPS24 acts as a tumor promoter in HCC progression.

## 3. Discussion

The latest literature reports that ribosomal proteins influence the progression and prognosis of HCC [7,18,19,20]. In addition to protein synthesis, RPS24 has role in tumor biology that has been neglected in research. In this study, we first described the presentation and prognosis of RPS24 in HCC and found that RPS24 can regulate HCC proliferation and immune infiltration in the tumor microenvironment, which might affect some signaling pathways, such as the E2F targets, G2M checkpoint, Wnt/β-catenin signaling pathway, and interferon-alpha/gamma. In addition, the biological functions of RPS24 in HCC cells were explored in vivo and in vitro. These results highlight that RPS24 might be a novel method of HCC management.

RPS24, one of the ribosomal proteins, plays a vital function in protein synthesis under physiological conditions. Surprisingly, studies found that RPS24 was highly expressed in prostate cancer [12] and colorectal cancer [21] and drove tumor progression. In addition, Zhang et al. found that RPS24 mRNA was elevated in the peripheral blood of HCC patients [22]. It was also found that RPS24 could promote cell proliferation and migration in human colon cancer [14]. However, in different types of tumors, the level of RPS24 expression is unknown. Here, the data mining of the TCGA dataset revealed that there was a high expression of RPS24 in 23 tumor tissues compared with para-cancerous tissues, especially in the case of HCC. In addition, we also validated this result using qRT-PCR based on HCC clinical cases. Moreover, a high expression of RPS24 is associated with various clinical parameters of the patients, such as AFP and the tumor size, and a poorer prognosis. This finding suggests that RPS24 might be a novel oncogene in these tumors, especially HCC.

To investigate the biological functions of RPS24 in HCC, we performed an enrichment analysis, and we found that RPS24 was closely associated with the pathways related to cell proliferation, such as DNA replication, the cell cycle, and cell cycle checkpoints. Subsequently, we also found that the down-regulation of RPS24 could impede the proliferation of the HCCM-3 and HUH-7 cells. To further verify the biological function of RPS24, we found that inhibiting the expression of RPS24 in HCC cells could inhibit the growth of subcutaneous tumor tissue in vivo. In addition, we discovered that highly expressed RPS24 could significantly enrich multiple cancer-promoting signaling pathways, such as the E2F targets, Wnt/β-catenin signaling, G2M checkpoint, and MYC targets, which accelerated tumor cell mitosis [23,24,25]. Furthermore, we found that the RPS24 hypermethylation or hypomethylation status affected the cancer cells’ malignant characteristics. However, the central signaling mechanism exerting the pro-cancer function of RPS24 in the tumor cells was unclear. Our findings suggest that RPS24 promotes the proliferation of HCC.

Stromal cells, especially immune cells, can influence tumor cell survival and proliferation via the enhanced recognition and killing of tumor cells [26]. In addition, an increasing array of evidence demonstrates that immune cells can be used to predict the prognosis of cancer patients [27]. Our study found that the infiltration levels of the DC cells, Tcm, Tgd, and Th17 cells were markedly decreased with high PRPS24 expression. For example, dendritic cells, the sentinels of the human immune system, precisely captured the slight difference between tumor cells and normal cells, thus enhancing the body’s anti-tumor immune system in HCC [28]. This suggests that RPS24 may restrict immune cell infiltration in the tumor microenvironment.

Immune checkpoint molecules can regulate the body’s immune system under physiological conditions to prevent autoimmune diseases. At the same time, tumor cells use immune checkpoint molecules to evade the surveillance of the immune system, allowing tumor cells to escape and grow [29]. In our study, highly expressed RPS24 induced the expression of some immune checkpoint genes, such as CTLA4, HAVCR2, and LAG3. Furthermore, today, immune checkpoint inhibitors are increasingly used in tumor therapy, and some significant breakthroughs have been made. For example, the CTLA4 monoclonal antibody has been shown to prolong the survival of HCC patients [30,31]. Moreover, we found that a high expression of RPS24 can predict a poor response to immunotherapy in HCC patients, which may not only be related to the regulation of immune cells and immune checkpoints but also be negatively related to the interferon-alpha/gamma signaling pathway [32]. Nevertheless, the mechanism has not been experimentally confirmed. Taken together, these findings suggest that RPS24 plays a significant role in determining the tumor immune status.

## 4. Materials and Methods

### 4.1. Data Acquisition and Preprocessing

Based on the TCGA GDC data portal (https://portal.gdc.cancer.gov/, accessed on 1 January 2020), the RNA-seq, clinal feature data (Table S3), and immune system infiltration of liver hepatocellular carcinoma (LIHC) were obtained, including 374 tumor samples and 50 normal tissues. According to the operation guide, the RNA-seq data with TPM normalization were used for the data analysis.

### 4.2. RPS24 Gene Expression and Its Relationship with Clinicopathologic Parameters

The mRNA levels of RPS24 in 28 tumors and normal samples were investigated using the ggplot2 package (version 3.3.3) in R (version 3.6.3). The correlation between RPS24 expression and the patients’ clinicopathological characteristics was evaluated using the Kruskal–Wallis Test and Dunn’s test, since the samples did not meet the normality threshold based on the normality test (*p* < 0.05). In addition, an ROC analysis was conducted to assess the predictive ability of RPS24. According to the median value of RPS24 expression, we divided the HCC patients into two groups, followed by a survival analysis. The impacts of the selected variables on the prognosis were evaluated via the univariate and multivariate Cox regression models.

### 4.3. DNA Methylation Analysis

The DiseaseMeth database (http://bio-bigdata.hrbmu.edu.cn/diseasemeth/index.html, accessed on 1 January 2020) enables researchers to conduct online studies of the promoter methylation status of the desired genes [33,34,35]. Therefore, the RPS24 promoter methylation status in HCC was explored based on the DiseaseMeth database. Additionally, the MethSurv database (https://biit.cs.ut.ee/methsurv/, accessed on 1 January 2020) was explored to reveal the effect of RPS24 on HCC patients’ survival [36].

### 4.4. Functional Enrichment Analysis of Differential Expression Genes (DEGs) and Micro (mi)RNA-Regulated Networks Analysis

To identify the differentially expressed genes (DEGs) between the high- and low-RPS24-expression groups, the R package DESeq2 was used with an adjusted *p*-value of <0.05 and |log2-fold change (FC) of | > 1.5. Then, the spearman correlation analysis between the top 10 DEGs and RPS24 was visualized using a heatmap. We further conducted functional enrichment analysis, including GO, KEGG, and GSEA [37].

Finally, we studied the regulation of miRNAs by RPS24 using miRWalk (http://mirwalk.umm.uni-heidelberg.de/, accessed on 12 May 2020) and the regulatory pathways and networks using ingenuity pathway analysis (IPA) [38,39,40].

### 4.5. Immune Cell Tumor Infiltration and Immune Checkpoints Analysis

The relationship between RPS24 and immune infiltration (immune cell and immune checkpoint molecules) was determined using the ssGSEA algorithm with the GSVA package (version 1.34.0) [41,42,43] and the TIMER database [44]. The potential response of each HCC patient to immunotherapy was predicted using the TIDE algorithm, comparing the RPS24-high-expressed and RPS24-low-expressed groups.

### 4.6. Patients and Tissue Specimens

All of the HCC and adjacent non-cancer samples were collected from Lanzhou University Second Hospital from May 2016 to May 2019, and the patients were diagnosed with HCC by pathological examination. Cohort 1: 20 pairs of fresh cancer samples and adjacent non-cancer samples were used to test the mRNA level of RPS24 by quantitative real-time PCR. Cohort 2: 86 pairs of cancer and adjacent non-cancer samples were formalin-fixed, embedded in paraffin, and used to detect the RPS24 protein expression using immunohistochemistry. The Ethics Committee of Lanzhou University Second Hospital approved the study (2020A-218). All the patients enrolled in our cohort signed informed consent.

### 4.7. Immunohistochemistry (IHC)

The operation process for the immunohistochemistry (IHC) analysis is referred to in our previous report [45]. The primary antibodies were anti-RPS24 (rabbit, 1:100, ABclonal Technology, Wuhan, China, A12123) and anti-Ki67 (rabbit, 1:7000, Proteintech, Wuhan, China 14831-1-AP).

The IHC scores were assessed independently by three pathologists. The staining area of RPS24 was quantified according to the proportion of stain-positive cells: 0 (0%), 1 (1–25%), 2 (26–50%), 3 (51–75%), and 4 (>75%). The staining intensity scores consisted of 0 (no staining), 1 (weak), 2 (moderate), and 3 (strong). The final staining scores were calculated using the intensity and percentage scores. Finally, tissue scores of ≥4 were defined as high expression, while the others were defined as low expression.

### 4.8. Cell Culture and Transfection

The human HCC cell lines HCCLM3 and HuH7 were collected from the Chinese Academy of Sciences Cell Bank, which were cultured with a medium (89% DMEM, 1% penicillin-streptomycin mixture, and 10% FBS) in 5% CO2 at 37 °C. To obtain HCC cells with RPS24 knockdown, lentivirus targeting RPS24 (Genepharma, Shanghai, China) was transfected when the cell density achieved 20–30%. The following shRNA sequences were used: sh-RPS24#1: 5′-CACCGGATGTCATCTTTGTAT-3′, and sh-RPS24#2: 5′-TCCCTGGATTATGCAAAGAAA-3′.

### 4.9. Quantitative Real-Time PCR (qRT-PCR)

The total mRNA of the HCC tissues and cells was extracted using Trizol reagent (Accurate Biology, code. AG11705). After spectrophotometrically measuring the RNA concentration of each sample, mRNA was obtained for cDNA synthesis using the EvoM-MLV RT kit (Accurate Biology, code. AG11705) and directly subjected to the next step of the quantitative PCR analysis using a CFX-96 real-time PCR system (Bio-Rad, Singapore). All the gene expression levels were normalized to the GAPDH gene using the 2^−ΔΔCt^ method, and gene-targeting primers were designed (Table 4).

### 4.10. Western Blot

Western blot analysis was applied to detect the target proteins according to the process reported by Zhang [46]. In this study, the primary antibodies were anti-RPS24 (1:1000, ABclonal Technology, Wuhan, China, A12123) and anti-GAPDH (mouse, 1:7000, Proteintech, Wuhan, China, 60004-1-Ig).

### 4.11. CCK-8 Assay and Colony Formation Assay

For the CCK-8 assay, stably transfected cells were placed on 96-well plates using the Cell Counting Kit-8 Reagent (Yeasen Biotechnology, Shanghai, China) every 24 h and incubated for 1 h. A microplate reader was used to measure the absorbance at 450 nm.

The stably transfected cells were seeded at a density of 500 cells/well for 14 days. Then, the colony formation of each well was calculated after cell fixation and staining.

### 4.12. Mouse Model

Male CB-17 SCID mice (*n* = 12) aged 5–6 weeks old were obtained from Beijing Vital River Laboratory Animal Technology and randomly classified into two groups. One group (*n* = 6) were inoculated with HCCLM3-shRNA control cells (2 × 10^6^) on the proper ventral back. Another group (*n* = 6) were subcutaneously inoculated with HCCLM3-shRNA-RPS24 cells (2 × 106) at the same site. The volume of the subcutaneous tumors was assessed every five days. Finally, tumor samples were collected for the following analysis after 20 days. The study was approved by the Ethics Committee of Lanzhou University Second Hospital (D2021-142).

### 4.13. Statistical Analysis

The statistical analysis of the data were performed using SPSS 20.0 software (IBM, Armonk, NY, USA) and GraphPad Prism version 8.0.2 (San Diego, CA, USA), and *p* < 0.05 was considered significant. One-way ANOVA/Student’s *t*-test and chi-square/Fisher’s exact test were used to analyze the quantitative and categorical data differences between the groups, respectively. The log-rank test was used to analyze the HCC patients’ survival.

## 5. Conclusions

In conclusion, this study demonstrated that RPS24 was highly expressed in HCC and associated with a poor prognosis through clinical data and bioinformatic analysis. RPS24 might affect tumor progression by regulating the biological behavior of cancer cells and the immune microenvironment.

## Figures and Tables

**Table 1:** Associations of RPS24 expression with the clinicopathologic parameters of HCC patients.

Characteristics	Total (N)	Odds Ratio (OR)	* p * -Value
Gender (Male vs. Female)	374	0.885 (0.573–1.365)	0.581
Age (>60 vs. ≤60)	373	0.629 (0.417–0.946)	0.026
T stage (T2, T3 and T4 vs. T1)	371	1.398 (0.930–2.106)	0.108
N stage (N1 vs. N0)	258	0.881 (0.104–7.439)	0.900
M stage (M1 vs. M0)	272	2.583 (0.326–52.588)	0.414
Pathologic stage (Stage III and Stage IV vs. Stage I and Stage II)	350	1.571 (0.969–2.570)	0.069
Tumor status (With tumor vs. Tumor free)	355	1.465 (0.962–2.239)	0.076
Histologic grade (G3 and G4 vs. G1 and G2)	369	3.044 (1.964–4.771)	<0.001
AFP(ng/mL) (>400 vs. ≤400)	280	5.060 (2.704–10.013)	<0.001
Child-Pugh grade (B&C vs. A)	241	1.279 (0.530–3.149)	0.583

**Table 2:** Immunohistochemical analysis showed the expression pattern of RPS24 in HCC tissues and adjacent non-tumor tissues.

RPS24 Expression	Tumor Tissue		Adjacent No-Tumor Tissues		*χ* ^2^	*p*-Value
	Cases	Percentage	Cases	Percentage		
Low	41	47.67%	55	63.95%	4.621	0.032
High	45	52.33%	331	36.05%		

**Table 3:** Relationship between RPS24 expression and clinical features of patients with HCC.

Variables	RPS24 Expression (*n* = 86)		*χ* ^2^	*p*-Value
	Low Expression Group (*n* = 41)	High Expression Group (*n* = 45)		
Age (years)			1.502	0.220
≥60	18	14		
<60	23	31		
Gender			2.851	0.091
Male	19	29		
Female	22	16		
ALT (U/L)			0.056	0.813
≥40	22	23		
<40	19	22		
AFP (ng/mL)			4.554	0.033
≥200	17	29		
<200	24	16		
LDH (U/L)			1.125	0.289
≥200	22	19		
<200	19	26		
Tumor size (cm)			4.508	0.034
≥3	23	35		
<3	18	10		
The number of tumors			5.454	0.020
>1	19	32		
≤1	22	13		
Tumor capsular			0.162	0.687
complete	20	20		
incomplete	21	25		
Degree of tumor differentiation			0.980	0.322
I + II	15	12		
III + IV	26	33		
Extrahepatic metastases			0.569	0.451
Positive	14	12		
Negative	27	33		
TNM staging			1.705	0.192
T1 + T2	24	20		
T3 + T4	17	25		

**Table 4:** The primers of targeted genes of this study.

Gene	Gene Sequences (5′-3′)
RPS24 (forward)	TCGGAAAGTGGCAAGCTGGTAAC
RPS24 (reverse)	CATTTCGGGCGGCTGTGAGAC
GAPDH (forward)	AGAGAAGAGCAAGCGAAATAC
GAPDH (reverse)	CCTTTCACTTCCTCCGATTAC

## Data Availability

The datasets used or analyzed during the current study are available from the corresponding author upon reasonable request.

## Supplementary Materials

The following supporting information can be downloaded at: https://www.mdpi.com/article/10.3390/ijms24010806/s1.
