# Supplementary material for: RPS24 Is Associated with a Poor Prognosis and Immune Infiltration in Hepatocellular Carcinoma
Source: Int J Mol Sci. 2023 Jan 2;24(1):806. doi: 10.3390/ijms24010806 (PMC9820840; doi:10.3390/ijms24010806)
Supplement: Supplementary file 1 [file ijms-24-00806-s001.zip › Figure S1.docx]

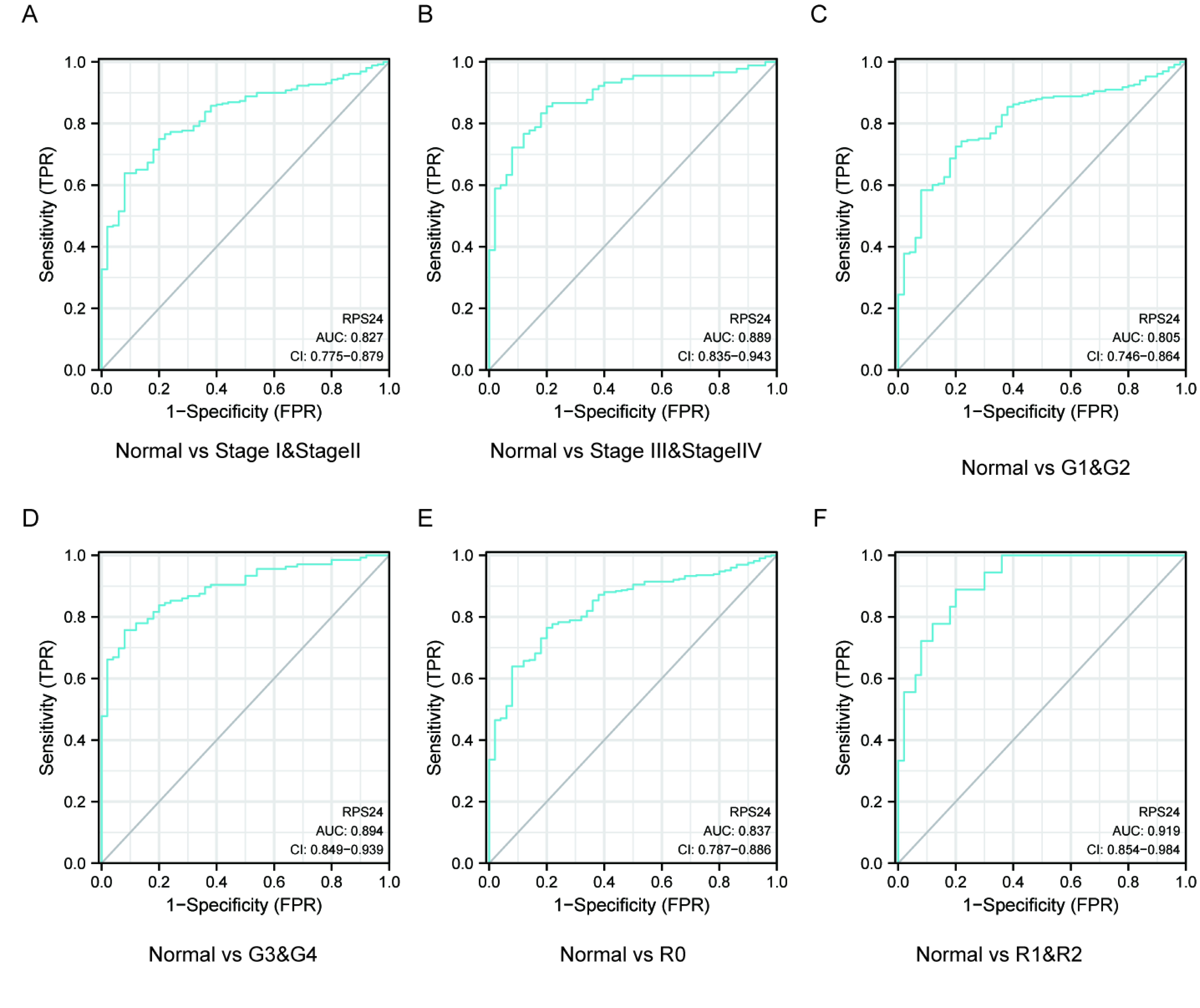


**Figure S1.** ROC curves of RPS24 mRNA expression in LIHC cohort using the TCGA dataset. ROC curves for classifying cancer patients with stages I&II(A), stages III&IV(B), G1&G2(C), G3&G4(D), R0(E), or R1&R2(F) versus normal individuals in the TCGA database.
