# Supplementary material for: RPS24 Is Associated with a Poor Prognosis and Immune Infiltration in Hepatocellular Carcinoma
Source: Int J Mol Sci. 2023 Jan 2;24(1):806. doi: 10.3390/ijms24010806 (PMC9820840; doi:10.3390/ijms24010806)
Supplement: Supplementary file 1 [file ijms-24-00806-s001.zip › Table. S1.docx]

Table S1 Relationship between different RPS24 transcripts and promoter methylation levels in HCC

| **Gene** | **genomic region** | **transcript** | **P-value** | **meanMethylDisea-meanMethylNormal** |
| --- | --- | --- | --- | --- |
| RPS24 | chr10:79791517-79794017 | NM_001026 | 0.000 | -0.015 |
| RPS24 | chr10:79791517-79794017 | NM_001142282 | 0.000 | -0.015 |
| RPS24 | chr10:79791517-79794017 | NM_001142283 | 0.000 | -0.015 |
| RPS24 | chr10:79791517-79794017 | NM_001142284 | 0.000 | -0.015 |
| RPS24 | chr10:79791517-79794017 | NM_001142285 | 0.000 | -0.015 |
| RPS24 | chr10:79791517-79794017 | NM_033022 | 0.000 | -0.015 |

meanMethylDisea-meanMethylNormal: positive numbers indicate high methylation levels in the tumor, and negative numbers indicate low methylation levels in the tumor.
