# Supplementary material for: RPS24 Is Associated with a Poor Prognosis and Immune Infiltration in Hepatocellular Carcinoma
Source: Int J Mol Sci. 2023 Jan 2;24(1):806. doi: 10.3390/ijms24010806 (PMC9820840; doi:10.3390/ijms24010806)
Supplement: Supplementary file 1 [file ijms-24-00806-s001.zip › Table. S2.docx]

Table S2 GO and KEGG pathway enrichment analysis on the DEGs of RPS24

| **ONTOLOGY** | **ID** | **Description** | **pvalue** | **p.adjust** | **qvalue** |
| --- | --- | --- | --- | --- | --- |
| BP | GO:0006614 | SRP-dependent cotranslational protein targeting to membrane | 5.3771E-16 | 2.8456E-12 | 2.5414E-12 |
| BP | GO:0006613 | cotranslational protein targeting to membrane | 2.0738E-15 | 5.4872E-12 | 4.9006E-12 |
| BP | GO:0045047 | protein targeting to ER | 3.3574E-14 | 5.9225E-11 | 5.2894E-11 |
| CC | GO:0022626 | cytosolic ribosome | 1.3212E-16 | 7.2933E-14 | 6.6758E-14 |
| CC | GO:0022625 | cytosolic large ribosomal subunit | 2.998E-12 | 8.2746E-10 | 7.574E-10 |
| CC | GO:0044445 | cytosolic part | 1.9986E-09 | 3.6774E-07 | 3.366E-07 |
| MF | GO:0005179 | hormone activity | 3.6648E-12 | 3.3277E-09 | 2.928E-09 |
| MF | GO:0048018 | receptor ligand activity | 1.1146E-11 | 5.0604E-09 | 4.4527E-09 |
| MF | GO:0001228 | DNA-binding transcription activator activity, RNA polymerase II-specific | 1.6148E-09 | 4.8874E-07 | 4.3004E-07 |
| KEGG | hsa03010 | Ribosome | 8.016E-11 | 2.3807E-08 | 2.2613E-08 |
| KEGG | hsa00140 | Steroid hormone biosynthesis | 5.8908E-10 | 8.7479E-08 | 8.3092E-08 |
| KEGG | hsa04080 | Neuroactive ligand-receptor interaction | 1.1435E-09 | 1.132E-07 | 1.0753E-07 |
| KEGG | hsa05204 | Chemical carcinogenesis | 8.3944E-09 | 6.2328E-07 | 5.9202E-07 |
| KEGG | hsa00830 | Retinol metabolism | 3.2908E-08 | 1.9547E-06 | 1.8567E-06 |
| KEGG | hsa04976 | Bile secretion | 2.6163E-07 | 1.295E-05 | 1.2301E-05 |
| KEGG | hsa00982 | Drug metabolism - cytochrome P450 | 3.7902E-07 | 1.6081E-05 | 1.5275E-05 |
| KEGG | hsa00980 | Metabolism of xenobiotics by cytochrome P450 | 1.4725E-06 | 5.4668E-05 | 5.1927E-05 |
| KEGG | hsa03320 | PPAR signaling pathway | 0.00038642 | 0.01275175 | 0.01211224 |

Abbreviations: GO: Gene Ontology; KEGG: Kyoto Encyclopedia of Genes and Genomes; BP: biological processes; CC: cellular components; MF: molecular functions.
