# Supplementary material for: RPS24 Is Associated with a Poor Prognosis and Immune Infiltration in Hepatocellular Carcinoma
Source: Int J Mol Sci. 2023 Jan 2;24(1):806. doi: 10.3390/ijms24010806 (PMC9820840; doi:10.3390/ijms24010806)
Supplement: Supplementary file 1 [file ijms-24-00806-s001.zip › Table. S3.docx]

Table S3. Baseline characteristics of HCC patients (n=374).

| **Characteristic** | **levels** | **Overall** |
| --- | --- | --- |
| n |  | 374 |
| Age, median (IQR) |  | 58 (49, 67) |
| Age, n (%) | ≤60 | 177 (47.3%) |
|  | >60 | 196 (52.7%) |
| T stage, n (%) | T1 | 183 (49.4%) |
|  | T2 | 85 (25.6%) |
|  | T3 | 80 (21.6%) |
|  | T4 | 13 (3.5%) |
| N stage, n (%) | N0 | 254 (98.4%) |
|  | N1 | 4(1.6%) |
| M stage, n (%) | M0 | 268 (98.5%) |
|  | M1 | 4 (1.5%) |
| Pathologic stage, n (%) | Stage I | 173(49.4%) |
|  | Stage II | 87(24.9%) |
|  | Stage III | 85 (24.3%) |
|  | Stage IV | 5 (1.4%) |
| Race, n (%) | Asian | 160 (44.2%) |
|  | Black or African American | 17 (4.7%) |
|  | White | 185 (51.1%) |
| Histological grade, n (%) | G1 | 55 (14.9%) |
|  | G2 | 178(48.2%) |
|  | G3 | 124 (33.6%) |
|  | G4 | 12 (3.3%) |
| Gender, n (%) | Female | 121 (32.4%) |
|  | Male | 253 (67.6%) |
| Tumor status, n (%) | Tumor free | 237 (99.2%) |
|  | With tumor | 2 (0.8%) |
| Residual tumor, n (%) | R0 | 327 (94.8%) |
|  | R1 | 17 (4.9%) |
|  | R2 | 1 (0.3%) |
| AFP(ng/ml), n (%) | ≤400 | 215 (76.8%) |
|  | >400 | 65 (23.2%) |
| Child-Pugh grade, n (%) | A | 219 (90.9%) |
|  | B | 21 (8.7%) |
|  | C | 1 (0.4%) |
| Fibrosis.ishak.score, n (%) | 0 | 75 (34.9%) |
|  | 1/2 | 31 (14.4%) |
|  | 3/4 | 28 (13.0%) |
|  | 5/6 | 81 (37.7%) |
| OS event, n (%) | Alive | 244 (62.4%) |
|  | Dead | 130 (37.6%) |

Abbreviations: AFP, alpha fetoprotein; OS, overall survival.
