# Supplementary figures and images for: RPS24 Is Associated with a Poor Prognosis and Immune Infiltration in Hepatocellular Carcinoma
Source: Int J Mol Sci. 2023 Jan 2;24(1):806. doi: 10.3390/ijms24010806 (PMC9820840; doi:10.3390/ijms24010806)

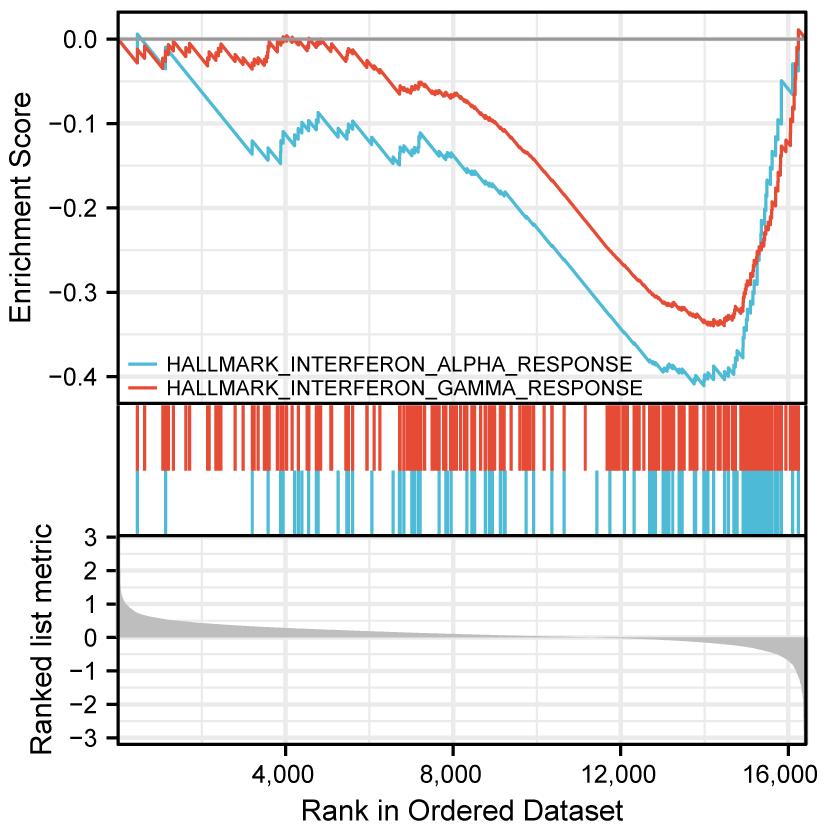

Supplement: Supplementary file 1 [file ijms-24-00806-s001.zip › Figure S2.tif]

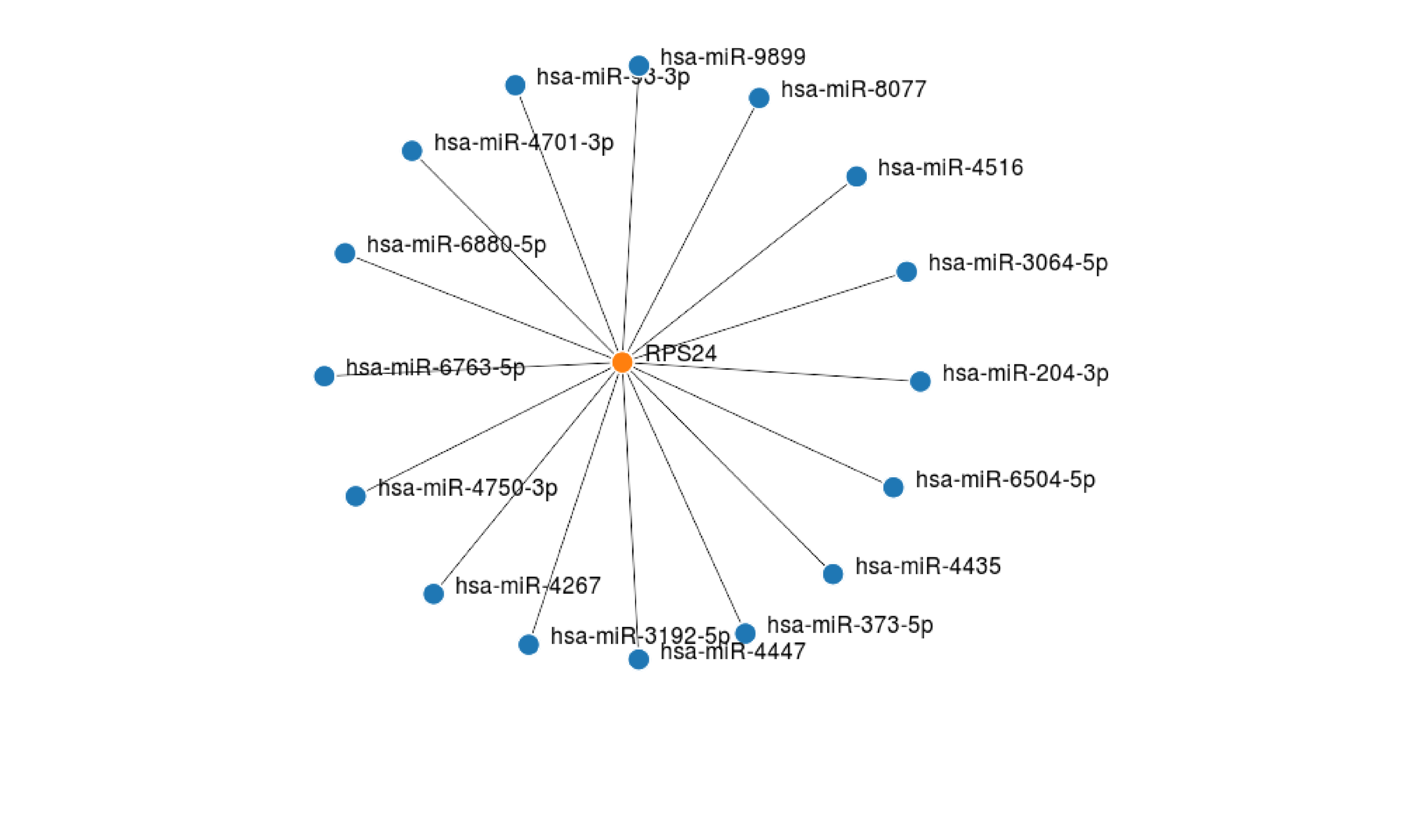

Supplement: Supplementary file 1 [file ijms-24-00806-s001.zip › Figure S3.tif]
